# A QSTR-Based Expert System to Predict Sweetness of Molecules

**DOI:** 10.3389/fchem.2017.00053

**Published:** 2017-07-25

**Authors:** Cristian Rojas, Roberto Todeschini, Davide Ballabio, Andrea Mauri, Viviana Consonni, Piercosimo Tripaldi, Francesca Grisoni

**Affiliations:** ^1^Instituto de Investigaciones Fisicoquímicas Teóricas y Aplicadas, CONICET, Universidad Nacional de La Plata La Plata, Argentina; ^2^Vicerrectorado de Investigaciones, Universidad del Azuay Cuenca, Ecuador; ^3^Milano Chemometrics and QSAR Research Group, Department of Earth and Environmental Sciences, University of Milano-Bicocca Milan, Italy; ^4^Kode s.r.l. Pisa, Italy

**Keywords:** sweetness, QSAR, molecular descriptors, classification, expert system

## Abstract

This work describes a novel approach based on advanced molecular similarity to predict the sweetness of chemicals. The proposed Quantitative Structure-Taste Relationship (QSTR) model is an expert system developed keeping in mind the five principles defined by the Organization for Economic Co-operation and Development (OECD) for the validation of (Q)SARs. The 649 sweet and non-sweet molecules were described by both conformation-independent extended-connectivity fingerprints (ECFPs) and molecular descriptors. In particular, the molecular similarity in the ECFPs space showed a clear association with molecular taste and it was exploited for model development. Molecules laying in the subspaces where the taste assignation was more difficult were modeled trough a consensus between linear and local approaches (Partial Least Squares-Discriminant Analysis and *N*-nearest-neighbor classifier). The expert system, which was thoroughly validated through a Monte Carlo procedure and an external set, gave satisfactory results in comparison with the state-of-the-art models. Moreover, the QSTR model can be leveraged into a greater understanding of the relationship between molecular structure and sweetness, and into the design of novel sweeteners.

## Introduction

Taste chemistry has become an important field of research for many disciplines, especially food chemistry. In fact, there exists a keen interest in research related to taste perception, since developments in molecular biology and biochemistry have provided the background for sweet-taste chemistry. Taste evocation is the result of soluble chemicals with different osmotic, endothermic and exothermic properties that interact with biological membranes on the taste buds on the tongue in different ways. Thus, the different tastes could be separated on the basis of the nature of such reactions; however, the mechanisms of how these interactions occur are not completely elucidated. Five accepted basic tastes exist: sweetness, bitterness, saltiness, sourness, and umami (also known as savory; Damodaran et al., 2008).

Li et al. (2002) described for the first time the sweet taste chemoreceptor, which is a G protein-coupled receptor (GPCR) constituted by the T1R2 and T1R3 subunits. This sweet chemoreceptor is able to recognize sweet stimuli produced by distinct sweeteners, such as carbohydrates, artificial sweeteners, amino acids, peptides, and proteins. Subsequently, Morini et al. (2011) proposed the use of the term “*receptor-mediated taste*” instead of “*basic taste*” due to the fact that the tastes are sensed by means of specific receptors and other mechanisms not necessarily mediated by the receptors. Thus, the human perception of these tastes varies from person to person, and it may be related to slight differences in psychology, anatomy, receptor function, concentration of the taste, or interaction with other substances.

Among the receptor-mediated tastes, sweetness is considered as the most important in a wide variety of foods, since it produces a pleasant sensation. Sucrose, the most common sweetener, is used as the international standard for measuring the sweetness of chemical compounds. Sucrose imprints a clean-sweet sensation without other aftertastes even at high concentrations, and it is obtained from economic renewable sources (sugar cane and sugar beets). Unlike the sweet taste, bitterness is usually perceived as an unpleasant receptor-mediated taste, although in some products such as tea, cocoa, coffee, beer, tonic water, or olives, the bitter taste is considered desirable. In this case, the quinine alkaloid is used as the standard for measuring relative bitterness. It is frequently used as a food additive. Finally, tastelessness could be defined as the lack of taste (insipid) or the loss of a perceived taste (e.g., sweet, bitter, sour, salty; Damodaran et al., 2008). Since both diabetic/dietetic medicines and foods should contain low-calorie sweeteners, preferably with a clean-taste, the pharmaceutical and food industries deal with the rational design and synthesis of potential compounds to be used as alternative sweeteners (Damodaran et al., 2008; Morini et al., 2011).

During the synthesis of new sweeteners, some variations in the chemical structure of a scaffold may change sweet molecules to non-sweet chemicals (tasteless, bitter, sour, and salty; Damodaran et al., 2008). In order to deal with this problem, scientist have been using approaches based on the Quantitative Structure-Activity/Property Relationships (QSAR/QSPR) to predict the sweetness of compounds to be synthesized. The QSAR/QSPR theory is an effective tool to build mathematical relationships between activities/properties of substances and their chemical structures, which is encoded by means of molecular descriptors (Todeschini and Consonni, 2009). Several Quantitative Structure-Taste Relationships (QSTRs) for predicting the sweetness of chemicals were proposed in the past years and are summarized in Table 1. The earlier work included compounds such as perillartine and aniline derivatives (Iwamura, 1980; van der Wel et al., 1987), sweet and bitter aldoxime derivatives (Kier, 1980), perillartine derivatives, aspartyl dipeptides, and carbosulfamates (Takahashi et al., 1982, 1984; Miyashita et al., 1986a,b; Okuyama et al., 1988), as well as sulfamate derivatives (Spillane and McGlinchey, 1981; Spillane et al., 1983, 1993, 2000, 2002, 2003, 2006, 2009; Spillane and Sheahan, 1989, 1991; Drew et al., 1998; Kelly et al., 2005). Moreover, two QSTR models to discriminate sweet, tasteless and bitter compounds have been proposed (Rojas et al., 2016a). Recently, Chéron et al. (2017) performed a predictive model for the discrimination of sweet and bitter molecules and the subsequent use of sweet compounds for predicting their relative sweetness (RS) property. In addition, some other recent studies remark the importance of the conformation-independent QSPR models for predicting the RS of sweet molecules (Rojas et al., 2016b; Ojha and Roy, 2017). Additionally, several recent scientific reviews regarding the applications of QSTRs are also available (Walters, 2006; Spillane and Malaubier, 2014; Rojas et al., 2016c).

**Table 1:** Summary of the performances of the QSTR classification models reported in the literature for predicting sweet taste of molecules.

**Models**	**Tastes**	**Classes**	**Method**	***d***	**N_train_**	**N_test_**	**NER_train_**	**NER_cv_**	**NER_test_**
Iwamura, 1980	Sweet and bitter	2	SLR	3	49	–^a^	–	–	–
Kier, 1980	Sweet and bitter	2	LDA	2	20	9	0.850	–	0.775
Spillane and McGlinchey, 1981	Sweet and non-sweet	2	Plot	2	35	12	0.914^b^	–	0.917^b^
Takahashi et al., 1982	Sweet and bitter	2	LLA	3	22	–	1	–	–
			*k*NN	6	22	–	0.909	–	–
Spillane et al., 1983	Sweet and bitter	2	LDA	3	33	–	0.807	–	–
Takahashi et al., 1984	Sweet and bitter	2	LDA	3	22	9	1	–	0.775
				2			0.955	–	0.775
Miyashita et al., 1986a	Sweet and non-sweet	2	SIMCA	4	50	–	0.798	–	–
Miyashita et al., 1986b	Sweet and bitter	3	SIMCA	5	91	–	0.840	–	–
Okuyama et al., 1988	Sweet and non-sweet	2	SIMCA	1^c^	25	–	0.868	–	–
					20	–	0.808	–	–
Spillane and Sheahan, 1989	Sweet and non-sweet	2	LDA	3	23	–	0.642	–	–
Spillane and Sheahan, 1991	Sweet and non-sweet	3	Plot	2	57	–	0.860	–	–
		2	LDA	3	33	–	0.848^b^	–	–
					23	–	0.870^b^	–	–
Spillane et al., 1993	Sweet and non-sweet (bitter, bitter followed by sweet aftertaste, sour and aniline- or hydrocarbon-like taste)	2	Plot	2	40	–	0.833	–	–
Drew et al., 1998	Sweet and bitter	3	DA	11^d^	50	–	1	–	–
Spillane et al., 2000	Sweet and non-sweet	2	LDA	4	101	–	0.665	–	–
			QDA			–	0.801	–	–
			CART	3		–	0.650	–	–
Spillane et al., 2002	Sweet and bitter	2	Plot	2	23	–	0.862	–	–
			LDA	4		–	0.850	–	–
			QDA			–	0.900	–	–
Spillane et al., 2003	Sweet and non-sweet	2	LDA	4	132	–	0.693	–	–
			QDA			–	0.683	–	–
			CART	3		–	0.815	–	–
Kelly et al., 2005	Sweet	3	LDA	8	75	8	0.547^b^	0.413^b^	0.500^b^
			QDA				0.773^b^	0.493^b^	0.250^b^
			CART classification				0.773^b^	–	–
			CART regression (R^2^ = 0.792)	7			0.813^b^	–	0.750^b^
Spillane et al., 2006	Sweet	3	CART classification	6	82	–	0.753	–	–
				7	82	–	0.580	–	–
				6	70	12	0.810	–	0.583^b^
			CART regression (*R*^2^ = 0.870)	7	70	12	0.807	–	0.909
Spillane et al., 2009	Sweet and non-sweet (bitterness, blandness or tastelessness)	2	LDA	2	58	–	0.655^b^	0.603^b^	–
		2	QDA	3	58	–	0.759^b^	0.603^b^	–
		2	CART	6	48	10	0.950	–	0.700
		3	CART	6	48	10	0.908	–	0.611
Rojas et al., 2016a	Sweet and tasteless	2	*k*NN	9	396	170	0.866	0.874	0.753
	Sweet and bitter			4	356	152	0.927	0.921	0.901
Chéron et al., 2017	Sweet and bitter	2	RF	5	796	191	0.997	–	0.902

*CART, classification and regression tree; d, number of descriptors; DA, discriminant analysis; kNN, k-nearest neighbors; LDA, linear discriminant analysis; LLA, linear learning machine; N_train_, number of molecules in the training set; N_test_, number of molecules in the test set; SIMCA, soft independent modeling by class analogy; QDA, quadratic discriminant analysis; RF, Random Forest; SLR, simple linear regression*.

a *Not available*.

b *Calculated as the ratio of correctly classified molecules to the total number of molecules (Accuracy)*.

c *Number of components for SIMCA analysis*.

d *Number of components considering for the DA analysis*.

The purpose of the work presented here was to build a QSTR-based expert system for the prediction of sweetness using a dataset of 649 molecules (435 sweet, 133 tasteless, and 81 bitter chemicals). To the best of our knowledge, this is the largest database of sweet chemicals ever used for predicting the sweetness of substances. The proposed expert system combines a structural similarity analysis and two QSTR models. Similarity structural analysis is based on extended-connectivity fingerprints (ECFPs), while the QSTR models are based on molecular descriptors (MDs) and N3 (*N*-nearest neighbors) and PLSDA (partial least squares discriminant analysis) classifiers. The proposed QSTR-based expert system was developed keeping in mind the five principles defined by the Organization for Economic Co-operation and Development (OECD) to make it applicable (OECD, 2007). The predictive ability of the model was properly evaluated by means of appropriate internal and external validation procedures. In addition, the chemical information of the molecular descriptors included in the QSTR models was interpreted and the model applicability domain properly defined.

## Materials and methods

### Experimental dataset and data curation

Each chemical compound can be experimentally associated with a predominant taste such as sweet, bitter, sour, and salty standards by trained panelists using a sip and spit method (Spillane et al., 1993, 2009). The initial experimental database, which is named TastesDB, was comprised of 727 chemicals retrieved from several scientific publications (refer to Table S1 for details of these publications). Each substance was associated with an experimental taste class (sweet, tasteless, or bitter). In this study, the tasteless and bitter categories were merged into a general non-sweet class, because the major scientific interest was in the identification of sweet compounds rather than bitter or tasteless chemicals. In fact, several studies on sweetness and taste have been conducted to discover and describe natural and synthetic sweeteners, sweetness potentiators and bitter blockers, to propose methods for characterizing different aspects of consumers' perception of sweetness. These perceptions are crucial aspects to be considered in order to improve the flavor, sweetness, texture, appearance, and physical properties in the development of food products (Damodaran et al., 2008).

The dataset was curated to remove molecules associated with wrong or problematic molecular structures, according to the following steps:

1. Pentadin, thaumatin, monellin, curculin, miraculin, brazzein, and mabinlin sweet proteins, were removed;
2. Disconnected molecular structures (salts), such as tripotassium glycyrrhizinate or aspartame-acesulfame salts, were retained;
3. For each molecule, the canonical Simplified Molecular Input Line Entry System (SMILES) strings were obtained from the designed molecular structure;
4. Tasteless and bitter classes were merged into a non-sweet class, as we wanted to focus on the prediction of sweetness vs. non-sweetness;
5. Compounds were merged according to their SMILES strings and then multiple-valued compounds were verified for the agreement between the annotated tastes:
  - Stereoisomers belonging to different taste classes (ambiguous molecules) were excluded (e.g., D-Arginine and L-Arginine, which are experimentally sweet and bitter compounds, respectively).
  - Amongst sweet molecules with the same SMILES strings, only one was retained (e.g., maltose and lactose).

The curated TastesDB dataset consisted of 649 molecules divided into two subsets of 435 sweet and 214 non-sweet (133 tasteless and 81 bitter) compounds, respectively (Table S1). QSTR studies regarding the prediction of the sweetness receptor-mediated taste were conducted by considering only homogeneous families of sweeteners (Iwamura, 1980; Kier, 1980; Spillane and McGlinchey, 1981; Takahashi et al., 1982, 1984; Spillane et al., 1983, 1993, 2000, 2002, 2003, 2006, 2009; Miyashita et al., 1986a,b; van der Wel et al., 1987; Okuyama et al., 1988; Spillane and Sheahan, 1989, 1991; Drew et al., 1998; Kelly et al., 2005), limiting their ability to predict the sweetness of other kinds of sweeteners. In order to generalize the predictiveness of the QSTR-based expert system, we used a dataset that covered a large chemical space of both sweet and non-sweet molecules. For example, derivatives of sucrose, abruside, acesulfame, isovanillic, mogroside, periandrin, saccharin, rebaudioside, cyclamate, suosan, aspartame, aspartyl dipeptides, glycyrrhizin, as well as several other heterogeneous compounds were included.

### Molecule representation

Structural characteristics of molecules were represented by means of both binary fingerprints and molecular descriptors. Binary fingerprints provide a holistic view of the molecular structure in terms of the presence/absence of identified molecular fragments. In particular, ECFPs (Rogers and Hahn, 2010) were used to represent molecular structures taking into account the information of the circular atom neighborhoods. ECFPs can be rapidly calculated and capture the common structural features of molecules by representing the presence/absence of particular substructures in a binary manner. For each molecule, a binary vector with 2,048 bits was calculated by using 2 bits per structural pattern and a maximum pattern length of 2.

In addition, classical molecular descriptors (MDs) were calculated, which are numbers that encode specific chemical/structural information of molecules (Todeschini and Consonni, 2009). The calculation of molecular descriptors on disconnected structures has been widely studied during the last years (Mauri et al., 2016). In the study presented here, the Dragon 7 approach (Kode srl, 2016) has been chosen, which consists of the application of the original definition and algorithm of the considered descriptors. If the original algorithm cannot be directly applied on disconnected structures, the Dragon approach provides a modification of the descriptor's original definition to allow the calculation since such modification is consistent with the theoretical sense of the descriptor.

In both cases, a two-dimensional molecular representation was selected instead of a geometrical representation to avoid irreproducible 3D structure optimizations. 3D descriptors could add valuable chemical information; however, since they require the geometrical optimization of molecules, the descriptor values can be affected by differences between 3D conformers with similar energies (Pearlman, 1998). In addition, the search of the minimum in the conformational energy hypersurface of molecules by means of an adequate optimization method involves high computational costs and long time. For this reason, the use of a conformation-independent molecular representation emerges as an alternative when dealing with the prediction of the sweetness and the relative sweetness (Rojas et al., 2016a,b; Chéron et al., 2017; Ojha and Roy, 2017).

### Multidimensional scaling

Multidimensional scaling (MDS; Kruskal, 1964) is a well-known multivariate method for unsupervised data exploration. MDS reconstructs similarities/dissimilarities between pairs of molecules by projecting data in a reduced hyperspace. In this way, data interpretation is facilitated. After the selection of a suitable number of dimensions to consider, a scatter plot of molecules provides a visual representation of the projected distances and can be used to analyze the relationships between chemicals as well as to identify clusters.

### Classification models

Since sweetness is a qualitative discrete response, classification approaches were used to establish mathematical relationships between the chemical/structural features of molecules and the modeled classes (sweet and non-sweet).

#### Partial least squares discriminant analysis (PLSDA)

PLSDA (Wold et al., 2001) is a well-known classifier that combines the properties of partial least squares regression (PLS2-based method) with the linear discrimination capability of a classification technique. In brief, this analysis finds relationships between the matrix of molecular descriptors and the class vector by calculating latent variables (LVs), which are orthogonal linear combinations of the original variables (descriptors). When dealing with PLSDA, molecular descriptors were autoscaled.

#### N-nearest neighbors (N3)

The recently proposed N3 classifier (Todeschini et al., 2015) is based on local molecular similarities. Thus, a molecule is classified by taking into account the class to which the most similar molecules (i.e., neighbors) belong. The neighbor contribution is weighted by the molecule similarity rank, whose role is modulated by an alpha parameter to be optimized. Range scaling and the average Euclidean metric were used when dealing with the N3 classifier.

The optimal number of latent variables (PLSDA) and the alpha parameter (N3) were optimized according to the lowest classification error in cross-validation.

### Reduction and selection of molecular descriptors

The V-WSP unsupervised variable reduction method (Ballabio et al., 2014) was used to reduce the presence of multicollinearity, redundancy, and noise in the initial pool of molecular descriptors. This method is a modification of the algorithm proposed by Wootton, Sergent, and Phan-Tan-Luu (WSP) for the selection of a subset of well-distributed points for design of experiments (DOE). In brief, V-WSP selects a subset of descriptors from the pool of candidates, in such a way as to have a minimal correlation from each descriptor in the defined multidimensional space. In addition, one of the fundamental steps of QSAR studies is the supervised selection of descriptors in order to build a parsimonious and predictive model based on a subset of informative descriptors. To this end, the Genetic Algorithms-Variable Subset Selection (GA-VSS) technique (Leardi and González, 1998) was coupled with both PLSDA and N3 classification methodologies in order to find the optimal subset of molecular descriptors. The essence of the GA-VSS is to start from an initial random population of chromosomes (i.e., models), which are binary vectors indicating the presence or absence of a given descriptor within the model. Then, an evolutionary process is performed and new chromosomes are generated by combination of chromosomes of the initial population (crossover) and/or random inclusion/exclusion of variables (mutation). If the new models have a reduced classification error, they are included in the population of chromosomes at the expenses of the worst ones, which are discarded.

### Model validation

Models were validated by means of an external test set constituted by 30% of the total number of molecules. Since the initial dataset was populated by a significant number of sweet substances, test molecules were randomly selected by maintaining the class proportion. Thus, the training set included 488 molecules (327 sweet chemicals and 161 non-sweet chemicals) and the test set was comprised of the remaining 161 molecules (108 sweet chemicals and 53 non-sweet chemicals). This partition guaranteed similar representation of the modeled classes. Training molecules were used for the supervised selection of molecular descriptors and the calibration of the QSTR-based expert system, while test molecules were used only to evaluate its prediction ability. A cross-validation protocol based on five cancelation groups divided in venetian blinds was used during the GA-VSS procedure (Ballabio and Consonni, 2013). The QSTR-based expert system was further validated by Monte Carlo (leave-many-out) random sub-sampling validation (Krakowska et al., 2016). The Monte Carlo approach defines many subsets by drawing samples in a random way from the available classes, based on a chosen number of iterations. Therefore, in each iteration, molecules were randomly divided into training (80%) and evaluation (20%) sets. The QSTR-based expert system was calibrated each time on the training molecules and then used to predict the class of evaluation molecules. The performance of the Monte Carlo validation was finally assessed by comparing the cumulative predictions vs. the experimental classes of test molecules.

Quality of the classification models was evaluated by means of sensitivity (Sn) and specificity (Sp) of classes (Ballabio and Consonni, 2013). Sensitivity of the sweet class was calculated as the ratio of the number of sweet compounds correctly classified to the total number of sweet compounds, while the specificity of the sweet class was calculated as the ratio of the number of non-sweet compounds correctly classified to the total number of non-sweet compounds. Since it is a two-class problem, the sensitivity of the sweet class corresponds to the specificity of the non-sweet class and *vice versa*. In addition, the non-error rate (NER) was calculated as the average of sensitivity values of sweet and non-sweet classes (Ballabio and Consonni, 2013). NER was used instead of Accuracy (which is the ratio of correctly classified molecules to the total number of molecules) to better estimate classification performance in the presence of unbalanced classes; non-sweet molecules are in fact less represented and constitute the 33% of the total number of molecules only.

### Software

HyperChem software (Hypercube Inc.)^1^ was used for representing the molecular structure, and the SMILES strings were obtained by using Babel software (O'Boyle et al., 2011). Molecular descriptors and extended connectivity fingerprints were calculated by means of DRAGON version 7 (Kode srl, 2016), while data curation and filtering of the dataset were carried out by means of a KNIME workflow written by the authors (Berthold et al., 2008). Data analysis and model calculations were performed in a MATLAB environment (MathWorks)^2^. The V-WSP variable reduction toolbox (Ballabio et al., 2014) was used to perform descriptors reduction, the classification toolbox for MATLAB (Ballabio and Consonni, 2013) was used for model calibration and the PCA toolbox for MATLAB (Ballabio, 2015) was used for both multidimensional scaling and molecular descriptors analysis. Genetic Algorithms variable subset selection was performed in MATLAB by means of code written by the authors. Classification toolbox and PCA toolbox are available at the Milano Chemometrics and QSAR Research Group website (http://michem.disat.unimib.it/chm/download/softwares.htm).

## Results and discussion

### Clustering sweet and non-sweet chemicals

**Figure 1:**
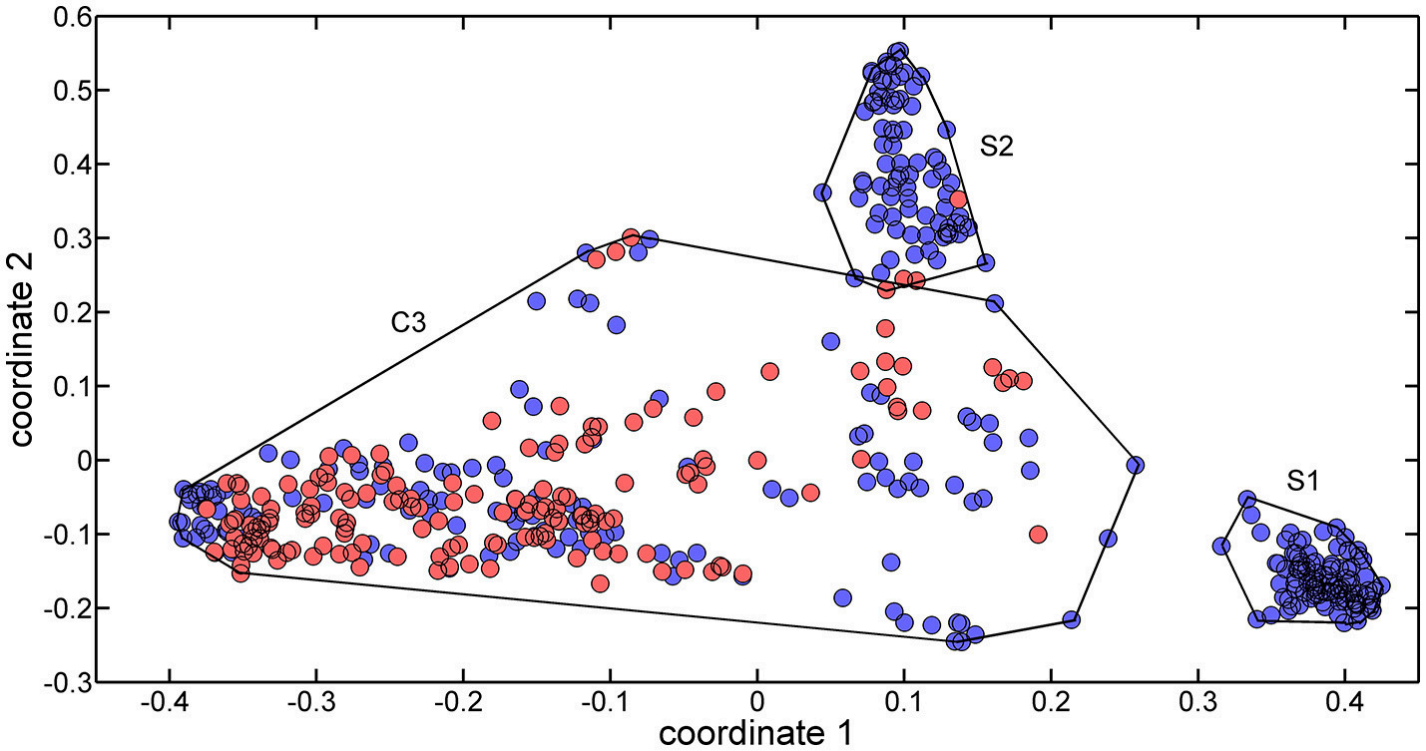
MDS plot of the two first coordinates (explained variance equal to 69.85%) for the training set molecules. Sweet molecules are marked with blue circles, and non-sweet molecules are market with cyan circles.

The 488 training molecules were initially used to perform a structural similarity exploratory analysis based on their extended connectivity fingerprints. To this end, molecular similarities were quantified by means of the Jaccard-Tanimoto similarity coefficient (Jaccard, 1912) and used to produce a multidimensional scaling (MDS) of the dataset. Figure 1 presents the MDS scores of the first two coordinates.

**Figure 2:**
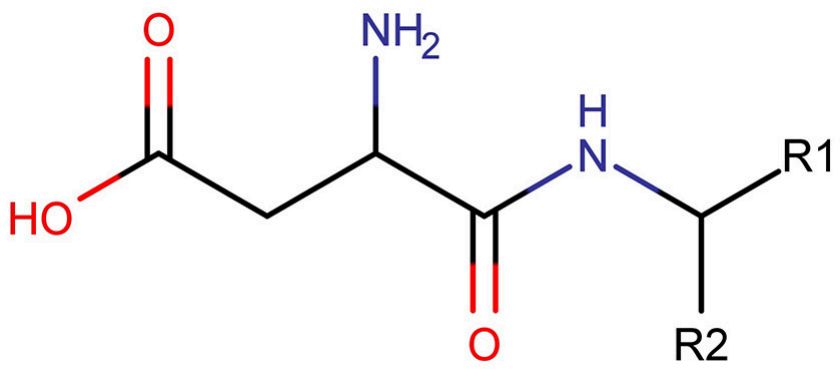
Common chemical scaffold of sweeteners grouped in cluster S1.

Three clusters (S1, S2, and C3) were identified in the MDS space, corresponding to three groups of molecules with specific structural similarities. Cluster S1 was comprised of 143 sweet molecules (Table S2), which have a common scaffold, as represented in Figure 2. The main characteristic of this molecular scaffold is the presence of the aspartic amino acid. However, other sweet chemicals with the same scaffold, but also containing benzene rings, are located in cluster C3, such as aspartame and N-(L-aspartyl)-1,1-diaminoalkane 5, along with some special cases of aspartyl derivatives (e.g., super aspartame, cyanoarylurea aspartame, aspartic acid fenchyl ester, and aspartame-acesulfame salt). The 107 molecules grouped in Cluster S2 (Table S3) included 100 sweet compounds (e.g., sucrose) and just 7 non-sweet compounds, such as the 6-Chloro-6-deoxy-D-galactose (tasteless), as well as a limited number of molecules exhibiting bitter taste (e.g., picrocrocin, methyl-α-D-2,6-dideoxy-gluco-pyranoside, methyl-α-D-3,6-dideoxy-gluco-pyranoside, methyl-α-D-4,6-dideoxy-gluco-pyranoside, and solanine). Finally, the remaining 399 chemicals and, in particular, the majority of non-sweet compounds are grouped in cluster C3 (Table S4).

Since the structural similarity analysis provided a satisfactory grouping of chemicals in terms of their taste, a QSTR-based expert system was considered as a suitable strategy to optimize the discrimination of sweet and non-sweet molecules. This system was structured as follows: the first step consisted of the identification of the cluster associated with a target molecule, using the ECFP-based structural similarity analysis; for example, if the molecule was assigned to cluster S1 or S2, it was likely to be predicted as sweet molecule. The second step consisted of the application of the QSTR models based on specific molecular descriptors which were calibrated using molecules included in cluster C3 to enhance the class discrimination in this chemical space.

### QSTR models based on molecular descriptors

The 297 training molecules belonging to cluster C3 were used to calibrate two different QSTR models based on the N3 and PLSDA approaches. The molecules were described by 3,763 conformation-independent Dragon descriptors. Descriptors with constant and near-constant values or those descriptors affected by missing values were excluded from the analysis. Moreover, to reduce the potential overfitting of the models due to highly correlated variables, the V-WSP unsupervised variable reduction approach was applied to further exclude another 1,255 descriptors at a correlation threshold of 0.95. The remaining 875 molecular descriptors were submitted to the subsequent supervised selection. This was carried out in two sequential steps: (1) GA-VSS (coupled with both N3 and PLSDA classifiers) was initially performed separately on each of the 18 blocks of molecular descriptors, and (2) the descriptors selected from each block were merged and a subsequent GA-VSS was carried out. The selection of the final sets of descriptors was performed by taking into account the NER classification parameter, as well as a balanced ratio between specificity and sensitivity of the sweet class. Two final models, each one based on six conformation-independent descriptors, were obtained with an optimal alpha of 1.5 for N3 and one latent variable (LV) for PLSDA.

The classification performance of the N3 model in fitting (NER = 0.748, Sn_sweet_ = 0.764, Sp_sweet_ = 0.732) and cross-validation (NER = 0.738, Sn_sweet_ = 0.750, Sp_sweet_ = 0.726), and the performance of the PLSDA classifier in fitting (NER = 0.722, Sn_sweet_ = 0.636, Sp_sweet_ = 0.809) and cross-validation (NER = 0.711, Sn_sweet_ = 0.607, Sp_sweet_ = 0.815) suggest a suitable capability of these models for predicting sweet taste inside cluster C3. The comparable performance in fitting and validation of the models indicate that these classifiers exhibit an overall balanced discrimination between the sweet and non-sweet classes with absence of potential overfitting. Descriptor details of the N3 and PLSDA models are shown in Table 2.

**Table 2:** Details of the conformation-independent Dragon molecular descriptors included in the N3 and PLSDA models in cluster C3.

**Name**	**Description**	**Block**	**Model**
F03[N-O]	Frequency of N—O at topological distance 3	2D Atom Pairs	N3
Uindex	Balaban U index	Information indices	
CATS2D_04_AL	CATS2D Acceptor-Lipophilic at lag 04	CATS 2D	
CATS2D_05_AL	CATS2D Acceptor-Lipophilic at lag 05		
C-026	R–CX–R	Atom-centerd fragments	
nCconj	Number of non-aromatic conjugated C(sp2)	Functional group counts	
F03[C-S]	Frequency of C—S at topological distance 3	2D Atom Pairs	PLSDA
MATS1s	Moran autocorrelation of lag 1 weighted by I-state	2D autocorrelations	
CATS2D_02_DN	CATS2D Donor-Negative at lag 02	CATS 2D	
CATS2D_04_AP	CATS2D Acceptor-Positive at lag 04		
ARR	Aromatic ratio	Ring descriptors	
D/Dtr07	Distance/detour ring index of order 7		

A graphical interpretation of the mechanistic effect of each descriptor in predicting the sweetness in the N3 models is not feasible because it is a local non-linear classifier; however, we attempted to explain the role of descriptors according to their chemical meaning. *CATS2D_04_AL, CATS2D_05_AL* (Renner et al., 2006) represent the frequency of hydrogen-bond acceptors and lipophilic atoms at a topological distance of 4 and 5 bonds, respectively. They indicate that sweetness of molecules may be attributed to the molecular hydrophobicity or the hydrophilic-lipophilic balance (HLB; Birch et al., 1994; Rojas et al., 2016a). Thus, the hydrophilic group works as an anchor allowing the fitting of the hydrophobic zone of the sweetener into hydrophobic binding sites in the sweet taste receptor (Yuasa et al., 1994). In fact, the presence of lipophilic atom pairs at a distance of 5 bonds (*CATS2D_05_LL*) already proved relevant in describing molecular relative sweetness (Rojas et al., 2016b). In addition, sweetness may also be influenced by the number of nitrogen and oxygen atom pairs (Carhart et al., 1985) at a topological distance of 3 bonds in the molecule (*F03[N-O]*) (Rojas et al., 2016a). Finally, the *nCconj* descriptor [number of non-aromatic conjugated carbon (sp^2^)], Balaban U index (Balaban and Balaban, 1991; which relates to the degree of branching of the molecule) and the number of aromatic carbons bonded to two aromatic carbon and one electronegative atom (O, N, S, P, Se, or halogens) (*C-026*) (Ghose et al., 1998) are also important for predicting the sweetness in the local non-linear N3 classifier.

**Figure 3:**
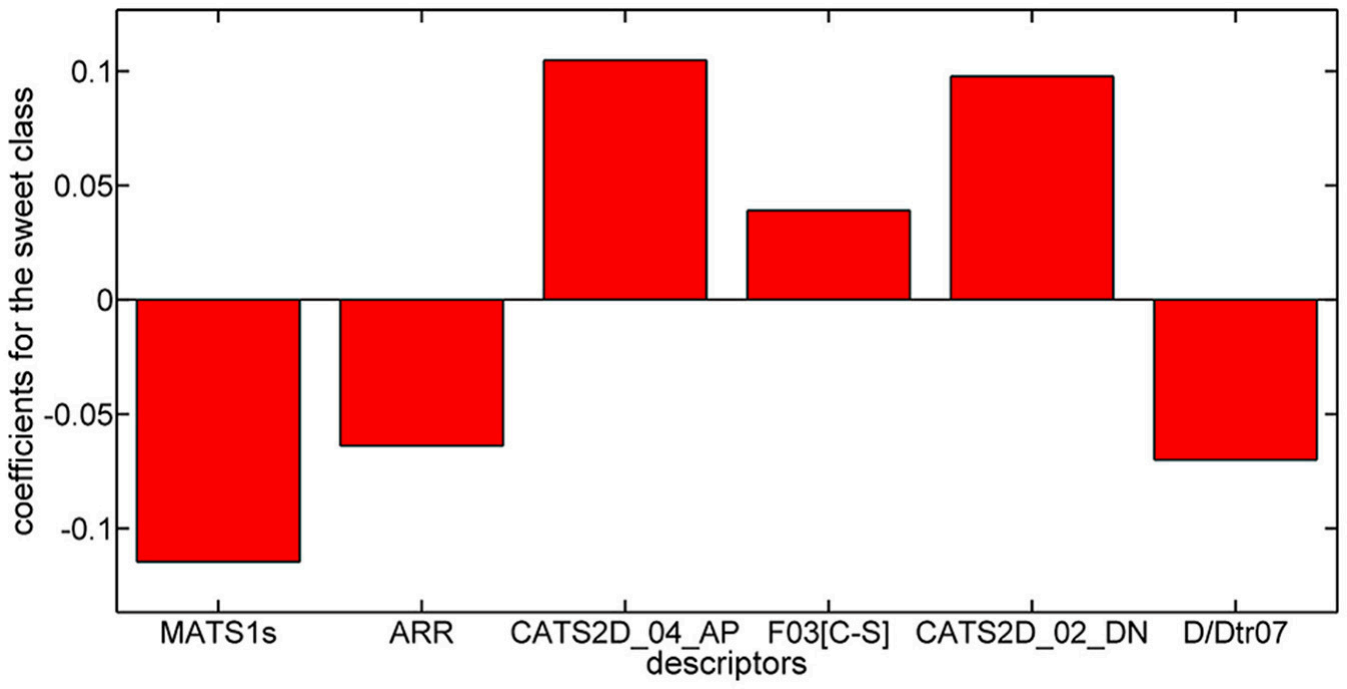
Coefficients for training descriptors in the PLSDA model for the sweet class.

Considering the PLSDA classifier, analysis of the model coefficients for the sweet class suggests that sweetness can be described by the *CATS2D_04_AP, CATS2D_02_DN*, and *F03*[*C-S*] descriptors. Figure 3 shows the coefficients of descriptors describing the sweet molecules. The selected *CATS2D* descriptors encode the presence of (1) pairs of hydrogen-bond donors (D) and negatively charged atoms (N) at a topological distance of 2 (*CATS2D_02_DN*) and (2) pairs of bond acceptors (A) (i.e., all N or O with at least one available lone pair electron) and positively charged atoms (P) separated by 4 bonds (*CATS2D_04_AP*). In fact, the presence of the positive-negative pharmacophores in the scaffold at a topological distance of 2 bonds was introduced for predicting the relative sweetness of molecules (Rojas et al., 2016b). *F03[C-S]* suggests that the sweetness is also related to the frequency of carbon-sulfur atom pairs in the skeleton at a distance of 3 bonds.

Coefficients for the non-sweet class of molecules have the same value but an opposite sign with respect to those of the sweet class. Thus, the descriptors associated with the non-sweet class correspond to the Moran autocorrelation of lag 1 weighted by I-state (*MATS1s*), the aromatic ratio (*ARR*) and the distance/detour ring index of order 7 (*D/Dtr07*). Moran autocorrelation of lag 1 weighted by I-state (*MATS1s*) is a descriptor calculated by applying the Moran coefficient (Moran, 1950) to the molecular graph by using the intrinsic state(s) as the atomic property. Positive values of the Moran coefficient produce positive spatial autocorrelations, whereas negative values of the coefficient are related to negative spatial autocorrelations. The distance/detour ring index of order 7 (*D/Dtr07*) (Randić, 1997) is a topological descriptor reflecting the ratio between the lengths of the shortest to the lengths of the largest through-bond paths between any pair of vertices belonging to 7-membered rings. The distance/detour ring in combination with other ring descriptors, such as the aromatic ratio (*ARR*) (i.e., ratio of the number of aromatic bonds to the total number of non-H bonds), indicates that non-sweetness is related to the presence of aromatic rings.

Since N3 and PLSDA models are based on different descriptors/modeling methods, a consensus analysis (Baurin et al., 2004) was applied in order to join information and predictions from these two sources. Individual models contain varying extents of noise (especially when dealing with large and heterogeneous datasets and noisy endpoints), which can be reduced by averaging the predictions of the models. The main assumption of *consensus* modeling is that the strengths of one model should compensate for the weaknesses of others models and *vice versa*. Therefore, each molecule was predicted only if the two QSTR models classified it in the same class; otherwise, it was not classified. The classification performance of the *consensus* approach in calibration (NER = 0.852, Sn_sweet_ = 0.792, Sp_sweet_ = 0.913, not assigned = 33%) and cross-validation (NER = 0.831, Sn_sweet_ = 0.772, Sp_sweet_ = 0.890, not assigned = 32%) confirms the main assumption of the *consensus* strategy by improving the overall prediction performance. On the other hand, the number of non-assigned molecules increased considerably. However, since the molecules of concern are those of cluster C3, the drawback of having non-assigned chemicals can be accepted in favor of increased classification performance.

### Assessment of the QSTR-based expert system

**Figure 4:**
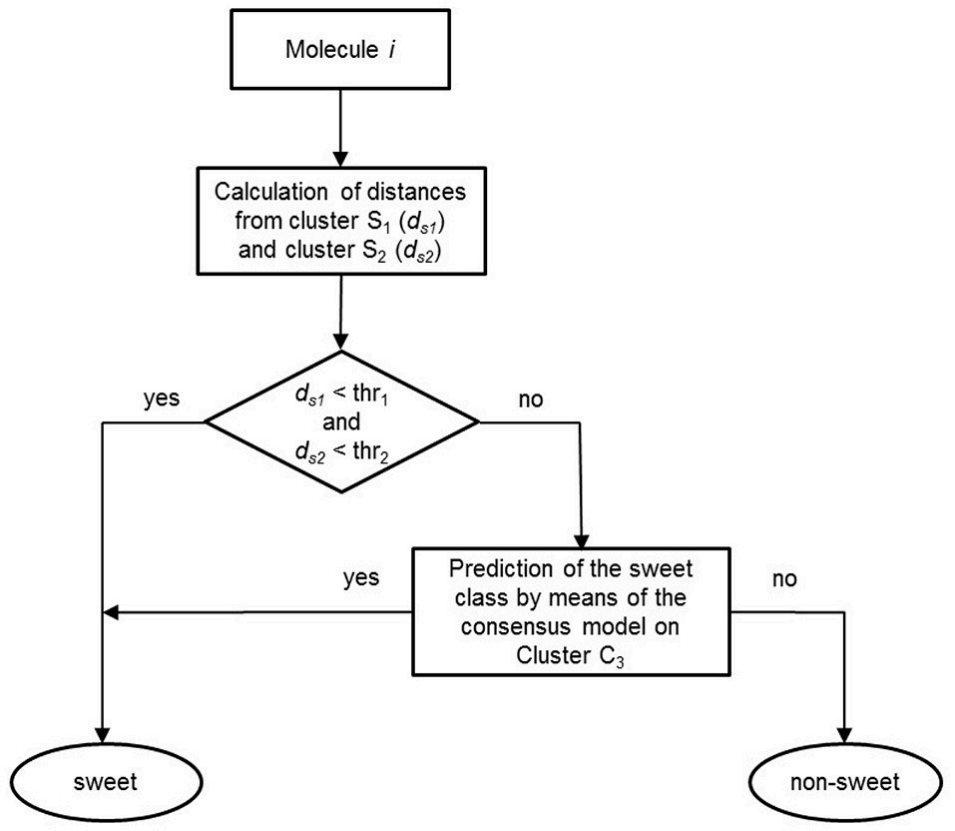
Workflow of the basic steps of the QSTR-based expert system for predicting the sweetness of chemicals.

Once the models were calibrated using the molecules of the C3 cluster, the QSTR-based expert system was assembled for the prediction of sweetness of the entire dataset. Figure 4 shows the structure of the proposed QSTR-based expert system. In particular, for any new target molecule, the sweetness prediction can be carried out on the basis of the following procedure:

1. Calculate ECFP vector for the target molecule and then its Jaccard-Tanimoto average distance to the molecules included in Clusters S1 (*d*_*s*1_) and S2 (*d*_*s*2_), respectively;
2a. If *d*_*s*1_ and *d*_*s*2_ are lower than defined thresholds (0.6 and 0.8, respectively), then the target molecule is classified as sweet, because of its high structural similarity to sweet molecules of clusters S1 or S2;
2b. Alternatively, if *d*_*s*1_ and *d*_*s*2_ are higher than the thresholds, then the target molecule is predicted by means of the consensus model based on the QSTR N3 and PLSDA classifiers.

**Figure 5:**
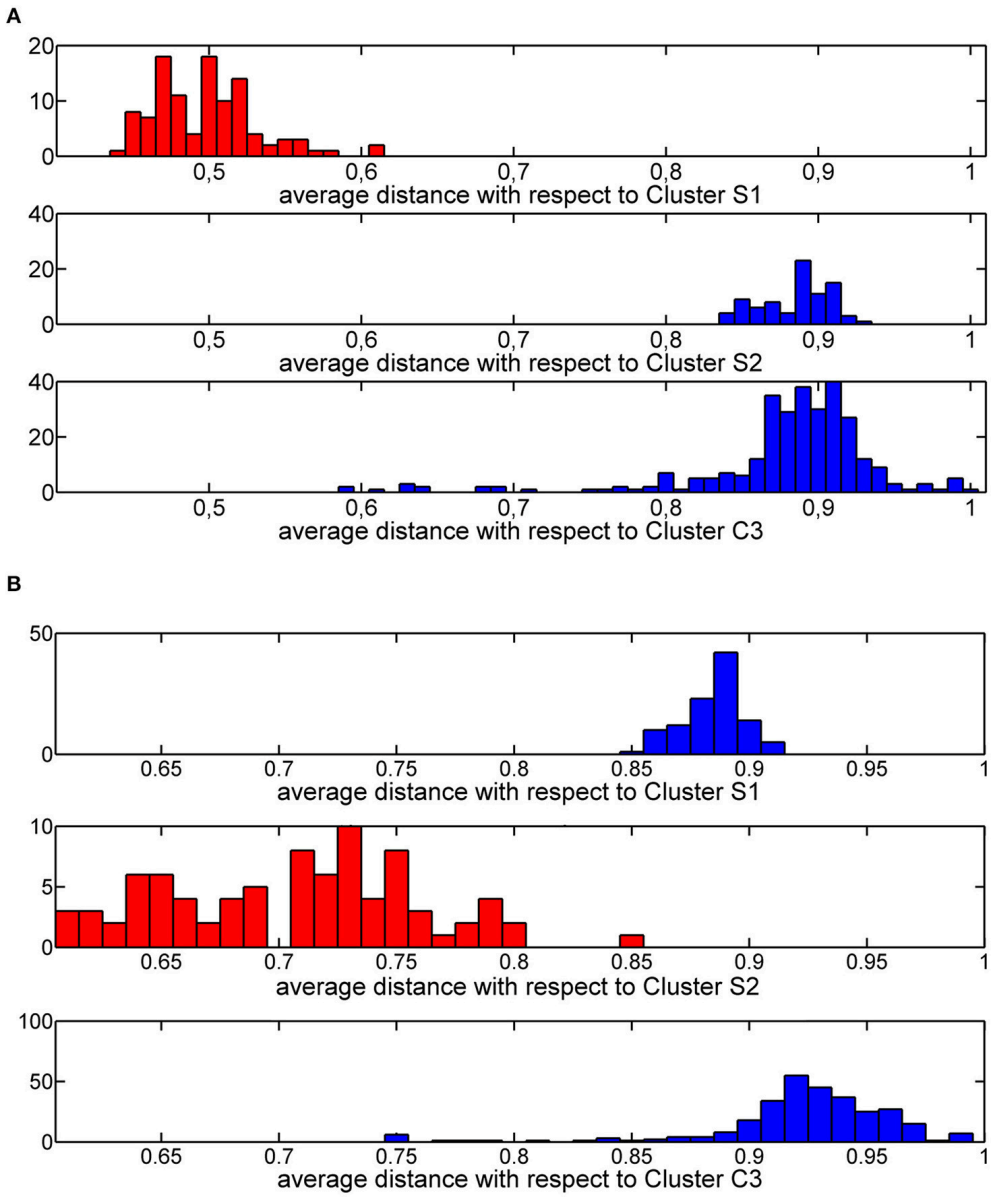
Histogram plot of the Jaccard-Tanimoto average similarity of the training molecules from molecules grouped in cluster S1 **(A)** and cluster S2 **(B)**.

The thresholds described in step 2a. were rationally chosen by analyzing the distribution of average similarities of each training molecule with respect to molecules of the three clusters. These distributions define a threshold value equal to 0.6 (Figure 5A) and a threshold value of 0.8 (Figure 5B) for cluster S1 and cluster S2, respectively.

Performance in classification of the QSTR-based expert system is listed in Table 3. Performance of the Monte Carlo validation based on 1,000 iterations (NER = 0.887, Sn_sweet_ = 0.927, Sp_sweet_ = 0.848, non-assigned = 20.5%) confirms the predictive power of the model. Finally, the 161 test molecules were used to assess the external predictive ability of the QSTR-based expert system. The results confirmed the predictive ability of the model (NER = 0.848, Sn_sweet_ = 0.880, Sp_sweet_ = 0.816, non-assigned = 19.3%). Model stability in fitting, validation and prediction, indicates that the proposed model does not exhibit potential overfitting, although the percentage of non-assigned molecules is c.a. 20%. Thus, the expert system presented in this paper could be useful to chemists who are dealing with the prediction of sweetness of both synthesized (virtual screening) and un-synthesized chemicals.

**Table 3:** Performance of the QSTR-based expert system based on the “*strict*” *consensus*.

	**NER**	**Sn**	**Sp**	**% not assigned**
Fitting	0.892	0.929	0.855	19.7
Monte Carlo	0.887	0.927	0.848	20.5
Test set	0.848	0.880	0.816	19.3

### Applicability domain assessment

Every QSTR prediction should be associated with a specific estimation of the applicability domain (OECD, 2007), in order to get an assessment of the prediction reliability. The applicability domain (AD) assessment of the QSTR-based expert system can be implemented on the basis of the following procedure:

1. Calculate ECFP vector for the target molecule and then its Jaccard-Tanimoto average distance to the molecules included in Clusters S1 (*d*_*s*1_) and S2 (*d*_*s*2_), respectively;
2a. If *d*_*s*1_ and *d*_*s*2_ are lower than defined thresholds (0.6 and 0.8, respectively), then the target molecule is inside the AD of the QSTR-expert model, because it can be assumed to be grouped together with molecules included in clusters S1 and S2;
2b. Alternatively, if *d*_*s*1_ and *d*_*s*2_ are higher than thresholds, the applicability domain assessment is carried out by comparing the leverage of the target molecule with respect to the leverage threshold for the PLSDA classifier; while an analysis of the distribution of average similarities is used for the N3 classifier.

Thus, any target molecule should satisfy one of these conditions to be inside the AD of the QSTR-based expert system, otherwise its sweetness prediction could be an extrapolation.

### Comparison and final discussion of the classification performance

The classification performance of both models included in the proposed QSTR-based expert system is considered appropriate, as well as the simplicity of the workflow of the expert system and the small number of molecular descriptors included in N3 and PLSDA models. The models presented in Table 1 from the existing literature were mainly calibrated by using small datasets and homogeneous sets of molecules, thus hampering the model generalization ability toward different types of chemicals (i.e., limited applicability domain). In addition, the majority of the studies did not perform validation of the QSTR models (Iwamura, 1980; Takahashi et al., 1982; Spillane et al., 1983; Miyashita et al., 1986b; Spillane and Sheahan, 1989, 1991). Thus, our QSTR-based expert system can be considered as a more general model for accurate prediction of sweetness of both un-evaluated and un-synthesized potential sweeteners exhibiting diverse scaffolds (i.e., a more general applicability domain). Additionally, this study provides the first QSTR model for sweetness prediction based on an expert system that (i) considers the use of both extended connectivity fingerprints and molecular descriptors and (ii) integrates the results from a structural similarity analysis along with *consensus* QSTR model predictions.

Several factors may affect the calibration of QSTR models for sweetness prediction such as the presence of unclear tastes of some sweeteners (i.e., multisapophoric or potential multisapophoric molecules). For instance, acesulfame potassium, sodium saccharin, hernandulcin, stevioside, and isocoumarin derivatives along with some sugar derivatives deliver bitterness in addition to sweetness. Their taste depends on the concentration of such molecules in solution (Birch et al., 1994). For molecules having more than two tastes, the taste perception may be complex (Shamil et al., 1987). For these reasons, humans are unlikely to discriminate these differences when dealing with multisapophoric molecules and this limitation may be due to the receptor saturation on the taste buds of the tongue or the polarization of the taste receptors (Birch et al., 1994).

On the other hand, sweeteners could exist in several equilibrium conformations that minimize their energy (Morini et al., 2011) and have more than one AH-B sites (Spillane and Sheahan, 1989; Damodaran et al., 2008); therefore, it is complex and difficult to define the active conformation and how such AH-B sites interact with the sweet-taste receptor to evoke the human sensation of sweetness. Moreover, the real interaction receptor-sweetener is not completely known. For instance, some compounds bind to the sweet receptor but they are not recognized as sweet (false positives), and other substances do not bind to the sweet receptor but are perceived as sweet (false negatives; Bassoli et al., 2008).

The simplicity and the satisfactory predictive ability of the QSTR-based expert system presented in this paper makes it a valid tool for scientists attempting to propose sweet molecular candidates either by synthesis or by virtual screening of very large available libraries. Thus, this model constitutes a starting point to understand the structure-taste relationships of molecules in which further evaluations could be addressed: (i) the conformational states of sweeteners, (ii) the mechanism of interactions between receptors and sweeteners (molecular docking and calculation of energies of binding), (iii) the measurement of the relative sweetness, and (iv) the identification of possible safety issues before using molecules as potential low-calorie sweeteners.

## Author contributions

CR and DB conceived the workflow, CR and FG curated the dataset, CR performed the calculations, and wrote the manuscript. All the authors contributed equally to the scientific planning, discussion and to the manuscript revision

### Conflict of interest statement

The authors declare that the research was conducted in the absence of any commercial or financial relationships that could be construed as a potential conflict of interest.

## Footnotes

^1^Hypercube Inc. “HyperChem”. http://www.hyper.com

^2^MathWorks “MATLAB”. http://www.mathworks.com

## Supplementary material

### Electronic supplementary material description

Supporting information for this work is presented in Table S1. This table shows the names, SMILES notations, experimental taste, and references of the curated TastesDB dataset, as well as the training and the test set assignments. In addition, molecule cluster assignments of the Multidimensional Scaling are reported in Tables S2–S4.
